# NEK2 enhances malignancies of glioblastoma via NIK/NF-κB pathway

**DOI:** 10.1038/s41419-022-04512-6

**Published:** 2022-01-14

**Authors:** Jianyang Xiang, Wahafu Alafate, Wei Wu, Yichang Wang, Xiaodong Li, Wanfu Xie, Xiaobin Bai, Ruichun Li, Maode Wang, Jia Wang

**Affiliations:** 1 Department of Neurosurgery, The First Affiliated Hospital of Xi’an Jiaotong University, Xi’an, Shaanxi 710061 China; 2 Center of Brain Science, The First Affiliated Hospital of Xi’an Jiaotong University, Xi’an, Shaanxi 710061 China

**Keywords:** CNS cancer, Nuclear receptors

## Abstract

Glioblastoma (GBM) is one of the most lethal primary brain tumor with a poor median survival less than 15 months. Despite the development of the clinical strategies over the decades, the outcomes for GBM patients remain dismal due to the strong proliferation and invasion ability and the acquired resistance to radiotherapy and chemotherapy. Therefore, developing new biomarkers and therapeutic strategies targeting GBM is in urgent need. In this study, gene expression datasets and relevant clinical information were extracted from public cancers/glioma datasets, including TCGA, GRAVENDEEL, REMBRANDT, and GILL datasets. Differentially expressed genes were analyzed and *NEK2* was picked as a candidate gene for subsequent validation. Human tissue samples and corresponding data were collected from our center and detected by immunohistochemistry analysis. Molecular biological assays and in vivo xenograft transplantation were performed to confirm the bioinformatic findings. High-throughput RNA sequencing, followed by KEGG analysis, GSEA analysis and GO analysis were conducted to identify potential signaling pathways related to NEK2 expression. Subsequent mechanism assays were used to verify the relationship between NEK2 and NF-κB signaling. Overall, we identified that NEK2 is significantly upregulated in GBM and the higher expression of NEK2 exhibited a poorer prognosis. Functionally, NEK2 knockdown attenuated cell proliferation, migration, invasion, and tumorigenesis of GBM while NEK2 overexpression promoted the GBM progression. Furthermore, High-throughput RNA sequencing and bioinformatics analysis indicated that NEK2 was positively related to the NF-κB signaling pathway in GBM. Mechanically, NEK2 activated the noncanonical NF-κB signaling pathway by phosphorylating NIK and increasing the activity and stability of NIK. In conclusion, NEK2 promoted the progression of GBM through activation of noncanonical NF-κB signaling, indicating that NEK2- NF-κB axis could be a potential drug target for GBM.

## Introduction

As is all known, glioblastoma (GBM) is one of the most common and aggressive malignant tumors in the central nervous system with a poor median survival time of only 12-15 months [1, 2]. Owing to the strong invasive ability of GBM and the resistance to radiotherapy and chemotherapy, it is difficult to prolong the survival time of patients with the current standard therapies, including surgical resection followed by radiotherapy and chemotherapy with temozolomide [3, 4]. Therefore, it is urgent to identify the biomarkers and drug targets associated with GBM progression to discover new therapeutic strategies [5].

NIMA-related kinase 2(NEK2), a member of the NIMA-related kinase family, is a poorly characterized serine/threonine kinase located in the centrosome [6, 7]. NEK2 activation results in the phosphorylation of centrosome proteins including NEK2-associated protein 1 (C-NAP1), rootletin, and β-catenin during the mitotic phase [8]. These proteins connect paired centrosomes during intermitosis. Subsequently, due to the phosphorylation caused by NEK2, they are dissociated from the centrosome, thereby promoting centrosome separation [9, 10]. In previous studies, it was demonstrated that the expression of NEK2 was significantly upregulated in a variety of malignant tumors, including breast cancer [11], colorectal cancer [12], cervical cancer [13], lung cancer [14] and glioma [15]. Moreover, NEK2 is correlated to various aspects of malignant transformation, including tumorigenesis, tumor progression and therapeutic resistance [16]. For instance, Xu T et al. reported that NEK2 activated the Wnt/β-catenin signaling pathway by increasing the expression of Wnt1, leading to the tumorigenesis and radioresistance in cervical cancer [13]. In another study, NEK2 was verified to be involved in the malignancy of GBM and the poor overall survival rate of GBM patients [17]. Despite these findings, the underlying molecular mechanism between NEK2 and GBM is yet to clarify.

The nuclear factor-kappa light chain enhancer of activated B cells (NF-κB), a family of evolutionarily conserved transcription factors, is an essential regulator of cell survival, proliferation, and differentiation [18]. There are five NF-κB family members in mammals, including NF-κB1 (p50/p105), NF-κB2 (p52/p100), RelA (p65), c-Rel, and RelB, which functions as dimers. Activation of NF-κB signaling is caused by either canonical or noncanonical pathways [19]. Canonical NF-κB pathway is mediated by p65/p50 heterodimers [20]. In unstimulated conditions, the heterodimers are inactive in the cytoplasm due to the mediation from the inhibitor of κB (IκB) family. Stimulation of inflammatory factors leads to the phosphorylation of various kinases and subsequently activates the inhibitor of κB kinase (IKK) complexes [20–22]. Activated IKK triggers the phosphorylation of IκB, which in turn causes the ubiquitination and proteasomal degradation of IκB and results in the release of p65/p50 heterodimer from the trimer. Thus, p65/p50 heterodimers translocate to the nucleus to regulate the transcription of related genes [23]. NF-κB-inducing kinase (NIK) is a crucial regulator in the noncanonical pathway. In the steady state, NIK is recruited to cIAP1/2 by binding to TRAF3, resulting in the decreased protein level of NIK via its ubiquitination and degradation [24]. Therefore, the NIK protein level is maintained at low levels. Stimulation of cytokines, such as TWEAK (TNF-like weak inducer of apoptosis) and CD40L causes the degradation of TRAF3, leading to the stabilization and accumulation of NIK. Subsequently, IKKα is phosphorylated and activated by NIK, which in turn phosphorylates p100. Phosphorylation of p100 triggers the proteolytic processing of p100 into p52 and the formation of transcriptionally active RelB-p52 complexes. After that, RelB-p52heterodimers translocate to the nucleus to regulate transcription [25]. Notably, previous studies verified that the NF-κB signaling pathway was closely related to tumorigenesis [26] and NF-κB signaling mediator genes are significantly overexpressed in GBM cells [27]. Therefore, it is essential to explore the potential molecular mechanisms of the NF-κB pathway in GBM.

In this study, we found that the expression of NEK2 is significantly upregulated in GBM and the higher expression of NEK2 exhibited a poorer prognosis. Moreover, NEK2 knockdown attenuated the malignancy of GBM cells and NEK2 overexpression promoted the GBM progression. In addition, we demonstrated that NEK2 activated the noncanonical NF-κB signaling pathway by interacting with NIK. All in all, we suggested that NEK2- NF-κB axis could be a potential drug target for GBM.

## Materials and methods

### Differential gene expression analysis

Gene expression datasets and relevant clinical information were extracted from public cancers/glioma datasets, including TCGA, GRAVENDEEL, REMBRANDT and GILL datasets. The limma package was then utilized for exploring the differentially expressed genes in these datasets [28]. The expression difference of individual gene was defined by log_2_ (Fold change) and adjusted *P* value, in which log_2_FC < −1 with an adjusted *P* value < 0.05 was defined as a upregulated gene.

### Ethical statement and human tissue samples

The usage of human tissue samples in this study was approved by the Scientific Ethics Committee at the First Affiliated Hospital of Xi’an Jiaotong University (approval no. 2016–18). All the tissue samples used in this study have obtained the necessary consent of the patients. Sixty-eight glioma tissue samples and five nontumor tissue samples were derived from patients that underwent surgical resection in the Department of Neurosurgery, The First Affiliated Hospital of Xi’an Jiaotong University from 2013 to 2019.

### qRT-PCR and RNA-seq

qRT-PCR assays were conducted as previously described [29]. RNeasy mini kits were used to extract total RNA according the manufacturer’s instructions. The concentration of RNA was determined by Nanodrop 2000. qRT-PCR was performed after synthesizing cDNA according to the standard protocols. GAPDH was utilized as an internal control. Relative mRNA expression was determined using the 2^−ΔΔct^ method. The primer sequences were shown as follows:

NEK2-F: 5′-ATCTCTAGAATGCCTTCCCGGGCTGAG3′ and NEK2-R:5′-ATACGGATCCCTAGCGCATGCCCAGGATC3′;

GAPDH-F: 5′-ACCCAGAAGACTGTGGATGG-3′ and GAPDH-R: 5′-TTCAGC TCAGGGATGACCTT-3′.

RNA-seq methodologies and initial analyses were conducted by LC Sciences using Illumina 2000 and 2 × 100 bp paired-end sequencing. The sequence results were obtained as FPKM (fragment per kilobase of exons per million reads) for each transcript.

### Western blot analysis

Western blot analysis was performed as previously described [30]. Antibodies used in this study were shown as below: Anti-β-actin primary antibody was purchased from Abcam and served as an internal control (ab115777). Anti-NEK2 primary antibody was purchased from Abcam (ab227958). Anti-p-IKKα/β (Ser176/177) primary antibody was purchased from Abcam (ab194528). Anti-p52 primary antibody was purchased from Thermo Fisher Scientific (MA5-17217). Anti-SNAIL primary antibody was purchased from Abcam (ab216347). Anti-SOX9 primary antibody was purchased from Abcam (ab185966). Anti-BMP2 primary antibody was purchased from Abcam (ab214821). Anti-NIK primary antibody was purchased from Abcam (ab216409). Additionally, Anti-Rabbit IgG (#7074) and Anti-Mouse IgG (#7076) were purchased from CST.

### Immunohistochemistry (IHC)

Immunohistochemistry was performed as previously described [31]. Anti-NEK2 primary antibody was purchased from Abcam (ab227958). Goat antirabbit IgG purchased from Abcam (ab97051) was used as the secondary antibody. Tissues embedded with paraffin were cut into 4-mm sections followed by deparaffinized, rehydrated, and stained with primary antibodies overnight at 4 °C. Afterward, the slides were incubated with corresponding secondary antibodies and stained with DAB. Tissues embedded with paraffin were cut into 4-mm sections. The sections were deparaffinized, rehydrated, and stained with primary antibodies overnight at 4 °C. The slides were subsequently incubated with secondary antibodies and stained with DAB. At last, hematoxylin was used to counterstain the slides and images were taken under the light microscope.

### Cell culture

GBM-derived primary culture cells were obtained from GBM patients underwent surgical resection in the Department of Neurosurgery, the First Affiliated Hospital of Xi’an Jiaotong University from 2013 to 2019. Fresh surgical GBM specimens were rinsed in PBS to remove adhering blood and visible necrotic portions. Then we carried out mechanical and enzymatic tissue dissociation using trypsin solution to obtain single-cell suspensions. The single cells were cultured in DMEM-F12 containing 15% fetal bovine serum (FBS), 2% B27 and antibiotics (1% penicillin and streptomycin) at 37 °C in a humidified atmosphere of 5% CO_2_. In addition, to identify the quality of these primary cells, we injected the primary cells into the brains of the nude mice. The primary cells which formed tumors were used for the next experiments.

A172, T98G, SF295, Normal Human Astrocytes (NHA) cell lines were purchased from Cell bank, Type culture collection, Chinese Academy of Sciences (Xi’an China). These cells were cultured in DMEM-F12 containing 10% FBS and antibiotics (1% penicillin and streptomycin) at 37 °C in a humidified atmosphere of 5% CO_2_.

### Lentivirus production and transduction

Lentivirus production and transduction were performed as described previously [31]. The lentiviral overexpression/knockdown NEK2 was designed and synthesized by Genechem (Shanghai, China). 1763 cells were transduced with NEK2 shRNA or scramble shRNA and 7209 cells were transduced with NEK2 overexpression lentivirus or vector control according to the manufacturer’s instruction. The transduced cells were selected with 0.5 mg/mL puromycin for 4 weeks. Lentiviral particles packaging the shRNA are targeting NEK2 #1 (5′- GCCATGCCTTTCTGTATAGTA-3′) and NEK2 #2 5′-CGTTACTCTGATGAATTGAAT-3′ the scramble control (5′- GCCAGAATTCTACCCTGGAAA-3′).

### Wound healing assays

The migratory ability of 1763 and 7209 cell lines was assessed by performing wound healing assays as previously described [32]. Briefly, the cells were seeded in six-well plates after adequately suspended. When the cells reached 90% confluence, a sterile P-200 pipette tip was used to produce a wound line in the center of the cell monolayer. The wound closure areas were visualized under inverted microscope at the same location at 0 or 24 h after seeding. The leading edges were marked by black lines and the relative distance of the borders was measure by Image J software.

### Matrigel invasion assays

The invasion ability of 1763 or 7209 cells was assessed by performing matrigel invasion assays as previously described [30]. 5 × 105 cells cultured in serum-free DMEM/F12 medium were sufficiently suspended and seeded into the upper chamber of 8μm transwell inserts (BD Biosciences, Franklin Lakes, NJ). DMEM/F12 medium with 10% fetal bovine serum (FBS) was added to the lower chamber. After incubation for 24 hours, cells adhering to the underside of the transwell membrane were fixed in 20% methanol and stained using 0.1% crystal violet. The non-migrated cells were slightly removed by cotton swabs from the upper surface of the filters. Subsequently, images were taken under inverted microscope.

### Sphere formation assay

Sphere formation assays were performed as described previously [33]. Cells were seeded into 24-well ultra-low attachment plates in serum-free DMEM/F12 medium with 5 µg/ml insulin, 0.4% bovine serum albumin, 10 ng/ml basic fibroblast growth factor, and 20 ng/ml human recombinant EGF. After incubation for 6 days, the size and number of spheres formed were visualized under inverted microscope.

### Colony formation assay

Colony formation assays were performed to verify the effect of NEK2 on the proliferation of GBM. 1763 cells and 7209 cells with different treatments were seeded into 6-well plates under indicating conditions. After cultured for 14 days to form colonies, these cells were fixed with methanol and stained with methylene blue.

### Flow cytometry

Flow cytometry assays were conducted as previously described [34]. Cell apoptosis was measured by the Alexa Fluor® 488 Annexin V/Dead Cell Apoptosis kit strictly following the manufacturer’s protocols.

### In vivo intracranial xenograft tumor model

Animals in this study was approved by the Ethics Committee of the School of Medicine, Xi’an Jiaotong University (approval no. 2016–085). All the nude mice in this study were chosen randomly. Six-week-old female nude mice were used for the intracranial xenograft tumor model. One thousand seven hundred and sixty-three and 7209 cells transduced with or without lentivirus (1 × 10^5^ cells in 2 uL PBS) were slowly injected into the brains of the nude mice as previously described [34]. Five mice were used for each group. The mice were sacrificed and perfused with ice-cold PBS and 4% (wt/vol) paraformaldehyde (PFA) when the following symptoms were observed: unsteady gait, arched back, more than 10% weight loss or leg paralysis. The mice brains were subsequently dissected and fixed in 4% PFA for 24 h, transferred to 10% formalin, and sectioned.

### Gene set enrichment analysis (GSEA) and Kyoto encyclopedia of genes and genomes (KEGG) analysis

Gene expression profiles were derived from the result of mRNA sequencing. All data were preprocessed by R software, including normalization and gene ID transformation. The limma package (PMID: 25605792) was used to identify differentially expressed genes in NEK2 knockdown group and control group. Next, the differently expressed genes (DEGs) were utilized to conduct gene ontology (GO) annotation and KEGG pathway enrichment analysis. The results are presented as a chord and a histogram respectively. Furthermore, GSEA was carried out according to User’s Guide (http://software.broadinstitute.org/cancer/software/gsea/wiki/index.php) to elucidate crucial pathways that are correlated with NEK2.

### Co-immunoprecipitation

Co-immunoprecipitation assays were performed as described previously [35]. The Pierce Co-Immunoprecipitation kit was purchased from Thermo Fisher Scientific. In brief, total proteins were extracted by IP lysis buffer. Subsequently, NEK2 antibody (1:50 final dilution) or NIK antibody (1: 50 final dilution) were added to the cell lysates. Nonspecific antibody IgG was set as a control. After incubation overnight at 4 °C, protein A Dynabeads (Thermo Fisher Scientific) was added and incubated with the cell lysates for 2 h. Then the beads were washed for three times with PBST and the pulled-down proteins were obtained. Western blot analysis was performed as previously described [30].

### Glutathione S-transferase (GST) pull-down assay

Briefly, GST-tagged NIK was transduced into HEK293T cells transduced with or without NEK2 overexpression lentivirus by lipofectamine according to the manufacturer’s instruction. After incubation for 24 h at 4 °C, cell lysates were collected and incubated with GST beads for 1 h. Subsequently, GST complexes were sufficiently washed with lysate buffer and subjected to western-blot analysis.

### Statistical analysis

All experiments were repeated independently for at least three times. The results in this study are presented as the mean ± standard deviation. Two-tailed *t*-tests were performed to evaluate statistical differences in two groups and one-way ANOVA analyses following Dunnett’s post-test were applied for comparisons between multiple groups. Kaplan-Meier survival analyses were conducted using log-rank test. All statistical analyses were carried out with SPSS 22.0 or GraphPad Prism 7 software and *P* < 0.05 revealed statistical significance.

## Results

### The expression of NEK2 is significantly upregulated in GBM patients

**Fig. 1:**
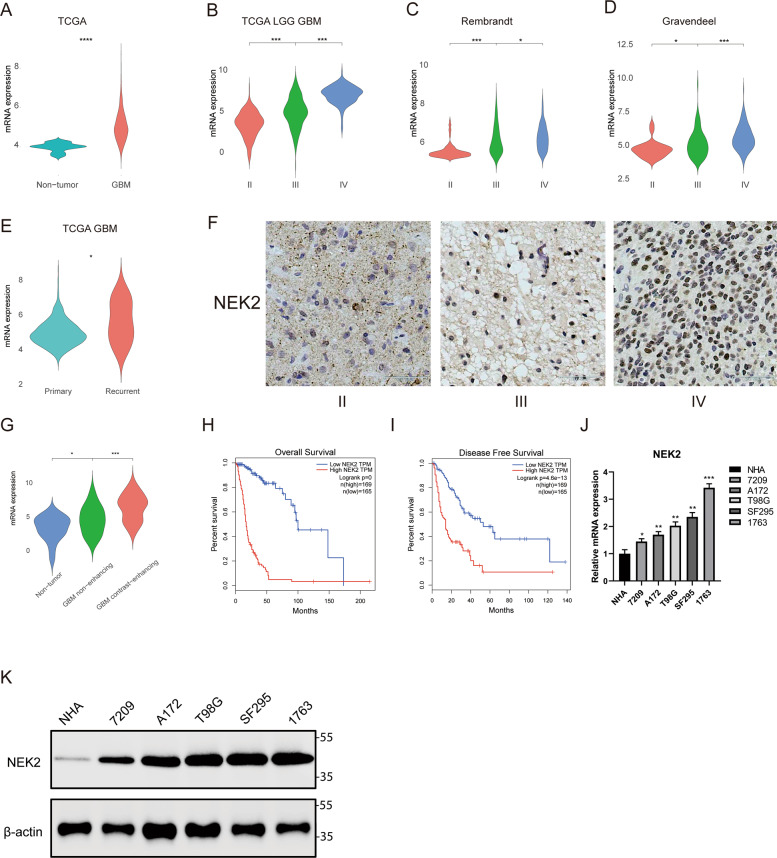
The expression of NEK2 is significantly upregulated in GBM patients. **A** Gene expression analysis in GBM versus nontumor by using TCGA database (*P < 0.001, with student’s *t*-test). **B** Gene expression analysis with TCGA database in different WHO grades of glioma (*P < 0.001, with student’s *t*-test). **C** Gene expression analysis with REMBRANDT databases in different WHO grades of glioma (*P < 0.001, with student’s *t*-test). **D** Gene expression analysis with GRAVENDEEL databases in different WHO grades of glioma (*P < 0.001, with student’s *t*-test). **E** Gene expression analysis for primary GBM and recurrent GBM by using TCGA database. **F** Representative IHC images of NEK2 in glioma samples. Brain tissue from epilepsy surgery was used as negative controls. **G** Gene expression analysis in contrast-enhancing GBM, nonenhancing GBM and nontumor by using GILL25114226 database. **H** Kaplan-Meier OS analysis for NEK2 expression by using GBM patient samples (*P = 0, with log-rank test). **I** Kaplan-Meier DFS analysis for NEK2 expression by using GBM patient samples (*P < 0.001, with log-rank test). **J** NEK2 mRNA expression in human primary glioma cell, commercial human glioma cell lines and NHA by qRT-PCR (**P < 0.01, *P < 0.05, with one-way ANOVA followed by Dunnett’s post-test). **G** Western blot analysis was conducted to detect the protein levels of NEK2 in human primary glioma cell, commercial human GBM cell lines and NHA. β-actin was set as an internal control. All data were reported as the mean ± SD of triplicate independent experiments.

To verify if *NEK2* is involved in the malignant progression of glioma, several public datasets were used to examine the mRNA expression level of NEK2 within different grades of glioma, including TCGA, GRAVENDEEL, REMBRANDT and GILL datasets. The results showed that the expression of *NEK2* was significantly increased in GBM compared with nontumor tissues (Fig. 1A). As is illustrated in Fig. 1B, C, D, *NEK2* expression levels increased considerably along with WHO grade progression. As is shown in Fig. 1E, NEK2 was upregulated in recurrent GBM, indicating that NEK2 associated with the recurrence of GBM patients. Moreover, the GILL database was used to investigate the expression of NEK2 in contrast-enhancing GBM, non-enhancing GBM and nontumor. It is obvious that NEK2 was upregulated in contrast-enhancing GBM (Fig. 1G). To gain a deeper understanding of the clinical significance of NEK2, IHC staining was performed by using samples derived from patients underwent surgical resection in the Department of Neurosurgery, The First Affiliated Hospital of Xi’an Jiaotong University from 2013 to 2019. The results indicated that NEK2 was overexpressed in GBM compared with low-grade glioma (Fig. 1F). Furthermore, Kaplan-Meier survival analysis from TCGA datasets demonstrated that glioma patients with higher expression of *NEK2* exhibited a shorter overall survival (Fig. 1H, I). In summary, *NEK2* showed significantly differential expression in correlation with malignant progress of glioma. Therefore, we picked *NEK2* as a candidate gene in this study. To gain a deep insight into the biological function of NEK2, we extracted and cultured GBM primary cells (1763 and 7209) from GBM patients underwent surgical resection. Afterwards, qRT-PCR and Western-blot were performed to detect the expression levels of NEK2 in primary cells, along with commercial human GBM cell lines and NHA. We confirmed that 1763 cells possessed a higher endogenous NEK2 expression level and 7209 cells possessed a lower endogenous NEK2 expression level. Consequently, we chose these two cell lines for the following experiments (Fig. 1J, K)

### NEK2 knockdown reduces the malignancy of GBM cells both in vivo and vitro

**Fig. 2:**
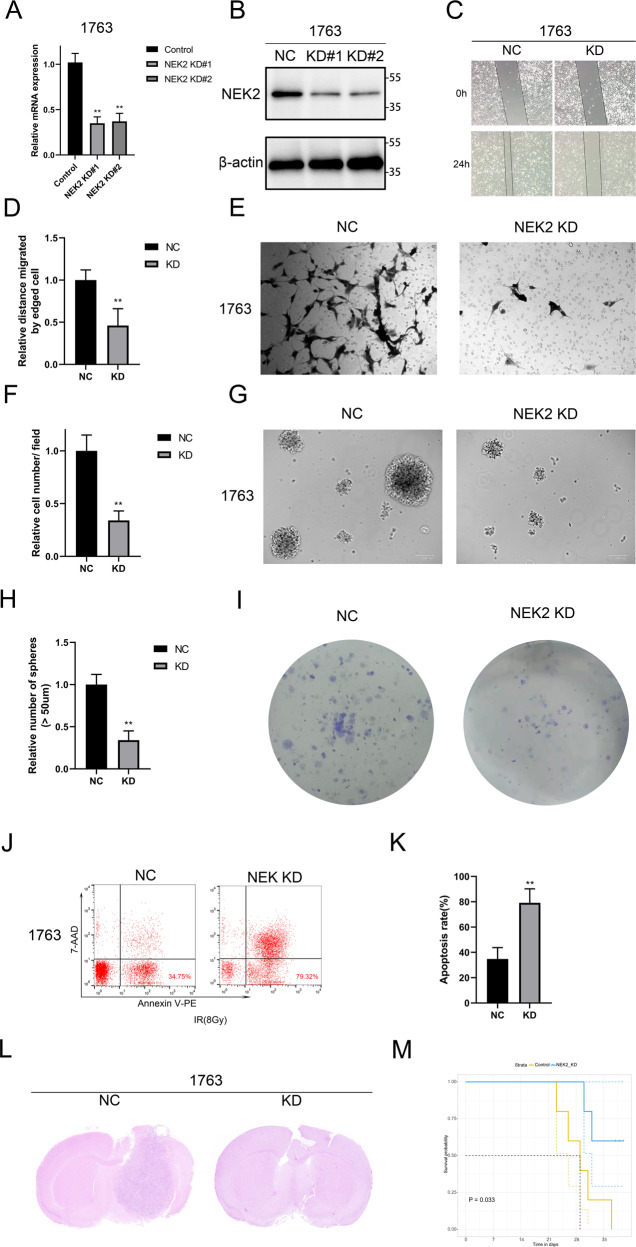
NEK2 knockdown reduced the malignancy of GBM cells both in vivo and vitro. **A** qRT-PCR analysis for the mRNA expression of NEK2 in 1763 cells transduced with lentiviral shNEK2 #1 and #2, and control group. **B** Western blot analysis for detecting the NEK2 protein expression in 1763 cell line transduced with lentiviral shNEK2 #1 and #2, and control. **C**, **D** Wound healing assays for exploring the effect of NEK2 knockdown on tumor malignancy in GBM cells. **E**, **F** Matrigel invasion assays were used to verify the inhibition of NEK2 knockdown on tumor invasive ability. **G**, **H** Sphere formation assays were performed to detect the sphere formation efficiency of NEK2 knockdown cells and control cells. **I** Colony formation assays to verify the effect of NEK2 knockdown on the proliferation of GBM. **J**, **K** Flow cytometry analysis using Annexin V and Propidium Iodide for apoptotic ratio analysis in 1763 cells transduced with lentiviral shNEK2 and negative control. **L** Representative H&E-stained images of mouse brain sections after the intracranial transplantation. Scale bars: 3 mm. **M** Kaplan-Meier survival curves of the xenograft mice in control group and NEK2 knockdown group (*P* = 0.033, with log-rank test). All data were presented as the mean ± SD of triplicate independent experiments.

To further investigate the function of *NEK2* in GBM, we transduced *shNEK2* lentivirus #1 and #2 to knockdown *NEK2* in 1763 cell line, which possessed a higher endogenous expression level of NEK2. qRT-PCR analysis showed that the mRNA expression of *NEK2* in 1763 transduced with shNEK2 lentivirus was significantly reduced when compared with shNT cells (Fig. 2A). Western-blot analysis was utilized to detect the protein level of NEK2. The results indicated that the protein level of NEK2 was also reduced by exogenous lentiviral knockdown (Fig. 2B). Moreover, we conducted wound healing assays and matrigel invasion assays to explore the effect of NEK2 knockdown on tumor malignancy in GBM cells. Obviously, the closure time was prolonged and the number of invasive cells was reduced in NEK2 knockdown cells when compared with control cells (Fig. 2C–F). Consistently, we performed sphere formation assays, as is revealed in Fig. 2G, H, the sphere formation efficiency of NEK2 knockdown cells was lower than that of the control cells. Meanwhile, colony formation assays were performed to verify the effect of NEK2 knockdown on the proliferation of GBM. Obviously, suppressing NEK2 attenuated the proliferation ability of GBM cells (Fig. 2I). Furthermore, flow cytometry assays were used to detect the apoptosis of NEK2 knockdown cells and control cells. The percentages of early (AV+; PI**−**) and late (AV+; PI+) cell apoptosis in NEK2 knockdown groups were remarkably increased (Fig. 2J, K). To evaluate the tumorigenicity of NEK2 knockdown cells in vivo, an orthotopic implanted xenograft mouse model was established. We observed that most of the mice transplanted with NEK2 knockdown cells failed to form tumors (Fig. 2L). In addition, Kaplan-Meier analysis showed that NEK2 knockdown increased the survival rate of xenograft mice, indicating that NEK2 knockdown inhibited tumor proliferation in vivo (Fig. 2M). Overall, these experiments indicated that NEK2 knockdown reduced the proliferation, migration, self-renewal and tumorigenesis of GBM cells both in vitro and in vivo.

### NEK2 overexpression accelerates GBM progression both in vitro and vivo

**Fig. 3:**
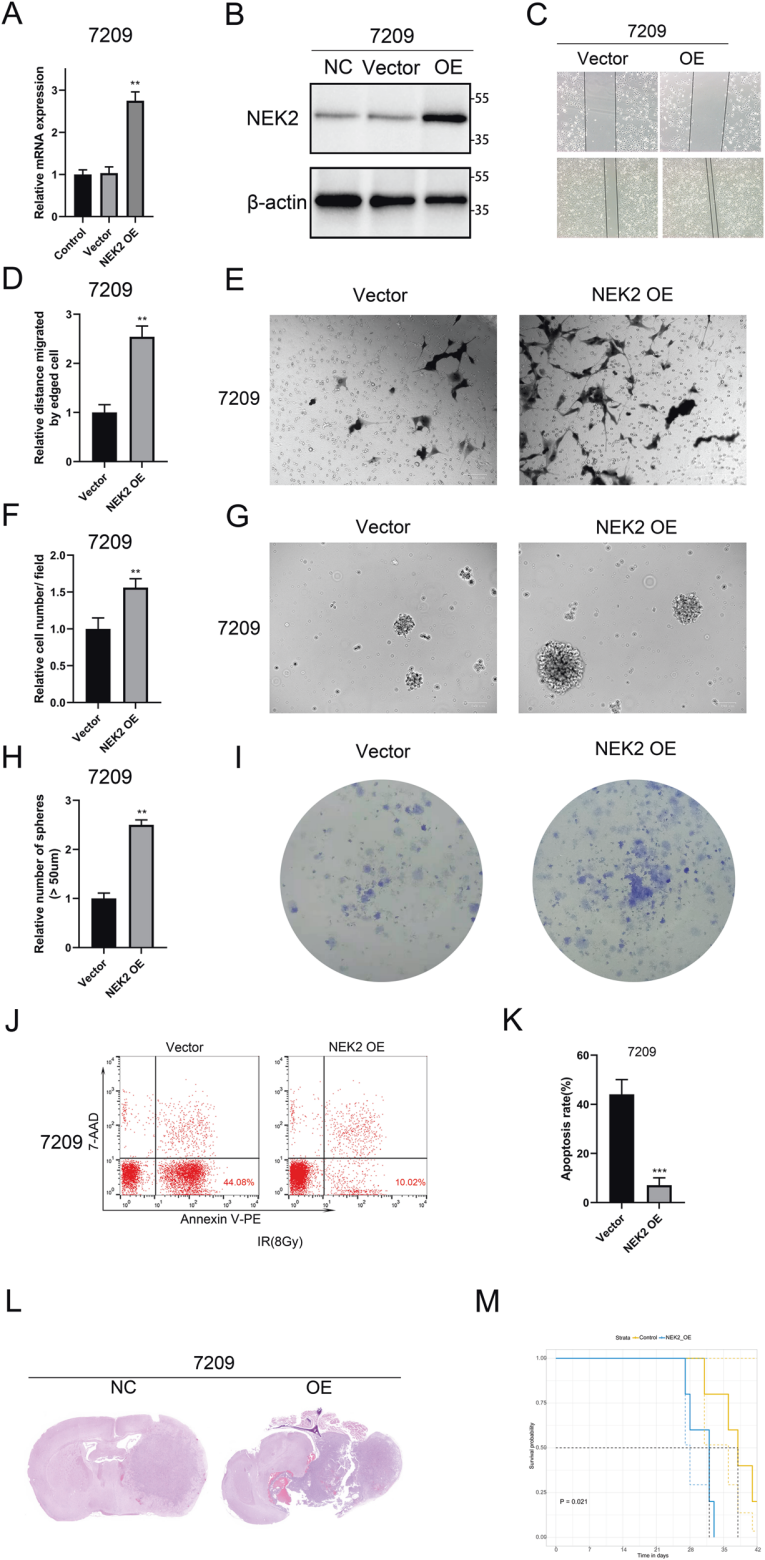
NEK2 overexpression promoted GBM progression both in vitro and vivo. **A** Analysis of the mRNA expression of NEK2 in pretreatment group and control group by qRT-PCR. **B** Western blot analysis for detecting the NEK2 protein expression in 7209 cells transduced with lentiviral NEK2, lentiviral vector and blank control. **C**, **D** Wound healing assays for exploring the effect of NEK2 overexpression on tumor malignancy in GBM cells. **E**, **F** Invasive ability of GBM cells transduced with lentiviral NEK2 and control. **G**, **H** Sphere formation assays were performed to detect the sphere formation efficiency of NEK2 overexpression cells and control cells. **I** Colony formation assays to verify the effect of NEK2 overexpression on the proliferation of GBM. **J**, **K** Flow cytometry analysis using Annexin V and Propidium Iodide for apoptotic ratio analysis in 1763 cells transduced with lentiviral NEK2 and control. **L** Representative H&E-stained images of mouse brain sections after the intracranial transplantation. Scale bars: 3 mm. **M** Kaplan-Meier survival curves of the xenograft mice in control group and NEK2 overexpression group (*P* = 0.021, with log-rank test). All data were presented as the mean ± SD of triplicate independent experiments.

To further confirm the role of NEK2 in glioma progression, exogenous NEK2 was introduced into 7209 cells via lentivirus transduction. qRT-PCR analysis showed that the mRNA expression of NEK2 was indeed increased in the pretreated 7209 cells (Fig. 3A). Consistently, Western-blot analysis demonstrated that the protein level of NEK2 was also increased in pretreated 7209 cells (Fig. 3B). To investigate the effect of NEK2 overexpression on the malignancy of GBM in vitro, we performed wound healing assays and matrigel invasion assays. As shown in Fig. 3C–F, the closure time was reduced and the number of invasive cells was increased in NEK2 overexpression cells when compared with the control group. Therefore, we proposed that NEK2 overexpression increased the proliferation and migration ability of GBM cells. Then we conducted sphere formation assays and consistently, the sphere formation efficiency of NEK2 overexpression cells was considerably increased than that of the control cells (Fig. 3G, H). Colony formation assays yielded similar results, which further confirmed this finding (Fig. 3I). In addition, we applied flow cytometry assays and found that the percentages of early (AV+; PI**−**) and late (AV+; PI+) apoptosis of NEK2 overexpressing cells were significantly lower than that of control cells (Fig. 3J, K). The intracranial xenograft tumor model was subsequently utilized to verify the promotion of NEK2 overexpression on the tumorigenicity of GBM cells in vivo. The results showed that NEK2 overexpression significantly increased the volume of tumor when compared with control mice (Fig. 3L). Additionally, NEK2 overexpression shortened the survival time of intracranial xenograft mice, as shown by Kaplan-Meier analysis (Fig. 3M). Altogether, these data revealed that NEK2 overexpression promoted the malignancy of GBM both in vitro and in vivo.

### NEK2 promotes gliomagenesis and the malignancy of GBM via activation of NF-κB signaling

**Fig. 4:**
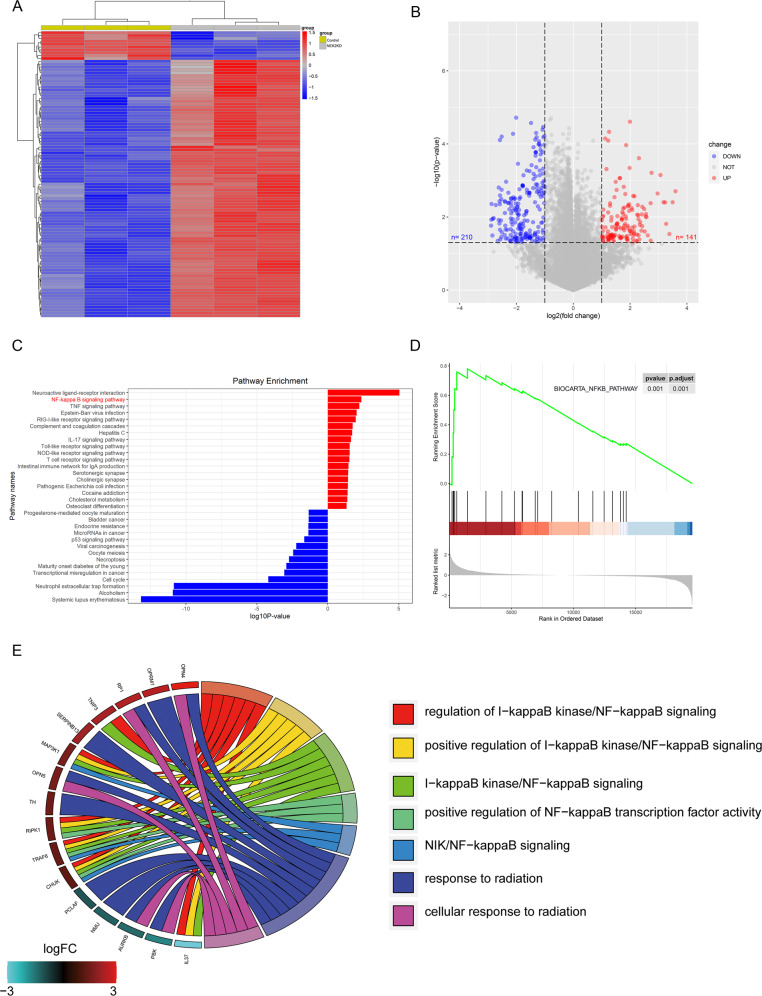
NEK2 promoted gliomagenesis the malignancy of GBM via activation of NF-κB signaling. **A** Hierarchical bi-clustering analysis was performed by using high-throughput RNA-seq data, indicating the significant gene signature in NEK2 knockdown 1763 cells compared with control 1763 cells. **B** Volcano plot for presenting the number of differentially expressed genes in NEK2 knockdown 1763 cells and control 1763 cells (log2FC > 1 or log2FC < −1, with an adjusted *P* value < 0.05). **C** Kyoto Encyclopedia of Genes and Genomes (KEGG) analysis for exploring the positive correlation pathways of NEK2. **D** Gene set enrichment analysis (GSEA) analysis showed the positive correlation between NEK2 and NF-κB signaling pathway. **E** Gene ontology (GO) analysis for differentially expressed genes in NEK2 knockdown group and control group.

As we have shown before, we suggested that NEK2 functioned as an oncogene in GBM. Therefore, it is critical to investigate the potential signaling pathways regulated by NEK2. We performed high-throughput RNA sequencing using three NEK2 knockdown samples of 1763 cells and three samples of control to identify differential expression genes. Hierarchical bi-clustering analysis indicated significant gene signatures in NEK2 knockdown 1763 cells compared with control 1763 cells (Fig. 4A). By performing differential gene expression analysis, we identified that 210 significantly downregulated genes in NEK2 knockdown group (log2FC > 1, adjusted *P* < 0.05) while 141 genes relatively upregulated in control group (log2FC < −1, adjusted *P* < 0.05; Fig. 4B). Afterward, we conducted Kyoto Encyclopedia of Genes and Genomes (KEGG) analysis to figure out the positive correlated pathways of NEK2. As is illustrated in Fig. 4C, we demonstrated that NEK2 was positively related to multiple molecular pathways of malignant tumors, including NF-κB signaling. Consistently, Gene set enrichment analysis (GSEA) analysis showed the significant positive correlation between NEK2 and NF-κB signaling pathways (Fig. 4D). Moreover, we conducted the Gene Ontology (GO) annotation by using those significant differential genes. The results indicated that NF-κB signaling was the enriched cancer-related GO terms (Fig. 4E). Thus, we picked NF-κB signaling pathway as the candidate downstream pathway of NEK2.

**Fig. 5:**
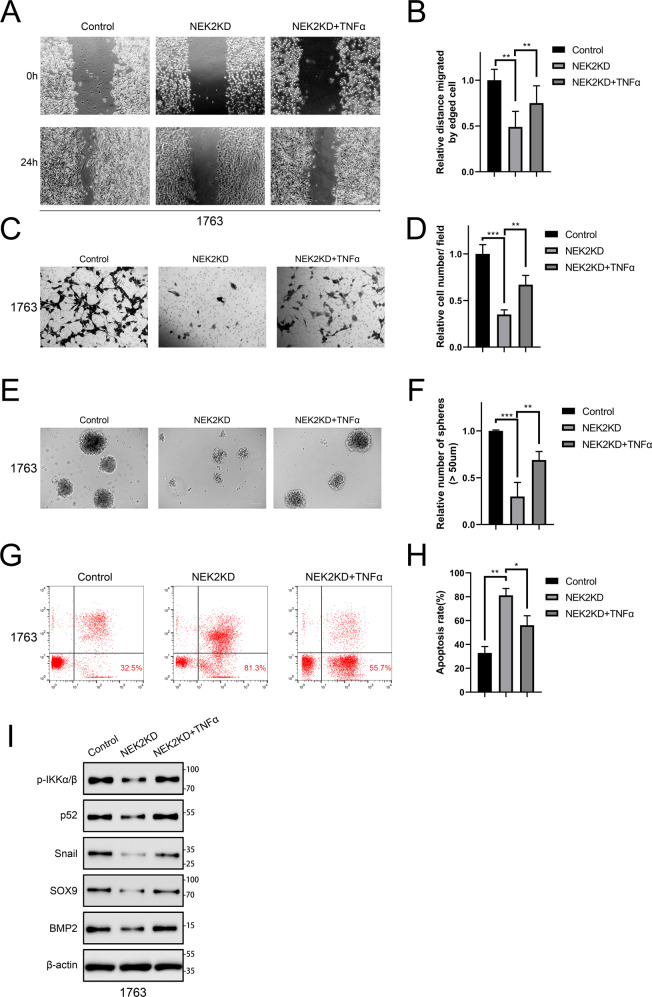
NEK2 promoted gliomagenesis and the malignancy of GBM via activation of NF-κB signaling. **A**, **B** Wound healing assays for exploring the effect of TNF-α on tumor malignancy in NEK2 knockdown GBM cells. **C**, **D** Matrigel invasion assays were used to verify the promotion of TNF-α on invasive ability in NEK2 knockdown GBM cells. **E**, **F** Sphere formation assays were performed to detect the sphere formation efficiency of NEK2 knockdown cells pretreated with TNF-α, NEK2 knockdown cells and negative control cells. **G**, **H** Flow cytometry analysis was carried out to measure the effect of TNF-α on the apoptotic ratio in NEK2 knockdown GBM cells. **I** Western blot assays were performed to detect the protein levels of the NF-κB signaling pathway as well as its downstream targets in NEK2 knockdown GBM cells pretreated with or without TNF-α.

According to a widely accept study, TNF-α regulated various signaling pathways in proliferation, apoptosis and inflammation via activation of NF-κB signaling. Therefore, we treated NEK2 knockdown 1763 cells with TNF-α. Subsequently, wound healing assays and matrigel invasion assays were conducted. As expected, the proliferation and migratory ability of GBM cells were significantly inhibited by NEK2 knockdown. However, TNF-α could partially reverse the inhibition (Fig. 5A–D). Sphere formation assays yielded similar results, which further confirmed this finding (Fig. 5E, F). In addition, flow cytometry assays were used to detect the apoptosis of NEK2 knockdown cells pretreated with or without TNF-α and control cells. Obviously, the percentages of early (AV+; PI**−**) and late (AV+; PI+) cell apoptosis in NEK2 knockdown GBM cells pretreated with TNF-α were significantly reduced when compared with NEK2 knockdown GBM cells (Fig. 5G, H). Taken together, these results suggested that NEK2 may promote the malignancy of GBM by activating NF-κB signaling pathway. To further evaluate the effect of NEK2 on NF-κB signaling activation, Western-blot assays were then performed. The results showed that NKE2 knockdown significantly reduced the protein levels of NF-κB signaling pathway, as well as its downstream targets, including Snail, SOX9, and BMP2, and by contrast, these landmark genes were partially improved on translation levels after TNF-α treatment was used (Fig. 5I). These findings indicated that NEK2 promoted the malignancy of GBM via activation of NF-κB signaling pathway.

### NEK2 activates the noncanonical NF-κB signaling pathway by phosphorylating NIK

**Fig. 6:**
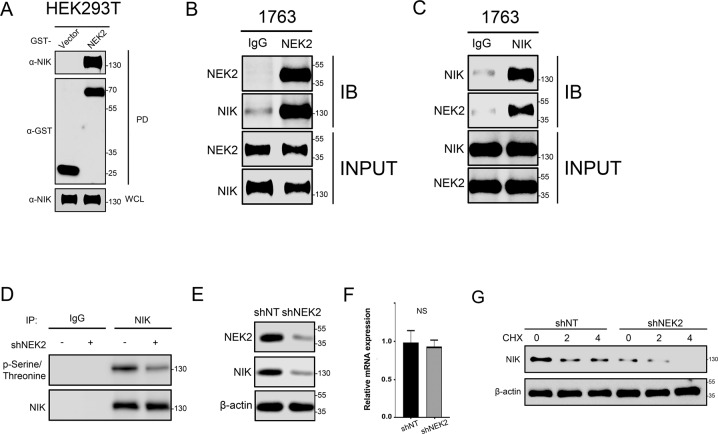
NEK2 activated noncanonical NF-κB signaling pathway by phosphorylating NIK. **A** GST pull-down assays were used to explore the interaction between NEK2 and NIK. **B** and **C** Immunoblotting (IB) for IP using a NEK2 antibody (**B**) or a NIK antibody (**C**). Nonspecific antibody IgG was used as a negative control. **D** Western-blot assays were used to detect the phosphorylation levels of NEK2 knockdown GBM cells and control GBM cells. **E** Western-blot assays for measuring the protein levels of NIK in NEK2 knockdown GBM cells and control GBM cells. **F** qRT-PCR analysis was performed to detect the mRNA levels of NIK in NEK2 knockdown cells and control cells. **G** The effect of proteasome inhibitor MG132 on protein level of NIK in GBM cells pretreated with or without shNEK2 lentivirus. β-actin was used as an internal control. All data were presented as the mean ± SD of triplicate independent experiments.

Our previous data indicated that NEK2 knockdown reduced the protein levels of p52, while noncanonical NF-κB signaling pathway is mediated by RelB-p52 heterodimers whose activation are dependent on NF-κB-inducing kinase (NIK). Therefore, we chose NIK as a potential downstream target of NEK2. To verify if NEK2 activates the noncanonical NF-κB pathway by acting on NIK, GST pull-down assays were performed. Exogenous NEK2 was introduced into HEK293T cell line via plasmid transfection. As shown in Fig. 6A, the results exhibited the presence of NEK2 and NIK in the affinity-purified complex, confirming that there was an interaction between NEK2 and NIK. CO-IP assays further validated this finding (Fig. 6B, C). Moreover, we used NIK specific antibody to obtain purified protein complexes of nontreated GBM cells and NEK2 knockdown GBM cells by immunoprecipitation. Nonspecific antibody IgG served as a negative control. Western-blot assays revealed that NEK2 knockdown significantly inhibited the phosphorylation of NIK (Fig. 6D). In addition, the protein levels of NIK were reduced in NEK2 knockdown GBM cells when compared with control GBM cells (Fig. 6E), indicating that NIK was an essential regulator through which NEK2 could activate NF-κB pathway. Moreover, we observed that the mRNA levels of NIK were not reduced in NEK2 knockdown 1763 cells when compared with control cells (Fig. 6F). This result suggested that the mRNA levels of NIK have no correlations with NEK2 and the regulation of NEK2 is post-translation. To confirm these findings, protein stability assays were performed with GBM cells transduced with or without shNEK2 lentivirus along with MG132, which was used to block the protein proteolysis. The results showed that the protein stability of NIK was significantly reduced in NEK2 knockdown GBM cells (Fig. 6G). Overall, our data so far above suggested that NEK2 phosphorylated NIK, which increased the activity and stability of NIK, thus inducing the activation of noncanonical NF-κB signaling pathway.

## Discussion

Glioblastoma (GBM) is one of the most lethal tumors in the human central nervous system [1, 2]. Current standard therapy consists of surgical resection followed by radiotherapy and chemotherapy with temozolomide. Despite the development of these clinical strategies over the decades, the 5-year survival rate of GBM patients is still less than 5% due to the strong proliferation and invasion ability and the acquired resistance to radiotherapy and chemotherapy [3, 4]. Previous studies identified that gene-targeted therapy for the molecular mechanism of tumorigenesis is gradually turning into a new direction for tumor therapy [5]. Therefore, developing new biomarkers and therapeutic strategies targeting GBM is in urgent need.

Accumulating evidence demonstrated that tumor-related kinases play a vital role in the progression, treatment resistance or prognosis of various tumors, and targeting kinase activity has been verified to be an effective way to reduce tumor growth [36–40]. NIMA-related kinase 2 (NEK2) is a poorly characterized serine/threonine kinase located in the centrosome, which regulates the centrosome separation and spindle formation, and ensures the stability and integrity of the chromosome structure. The ectopic expression of NEK2 leads to the premature separation of centrosomes, enhancement of centrosome amplification, chromosome instability (CIN), and aneuploidy [9, 10]. Accumulating evidence reveals that NEK2 is upregulated in a variety of cancer tissues and cell lines, indicating the involvement of NEK2 in tumorigenesis [11–15]. For instance, a recent study demonstrated that NEK2 regulated the expression of KDM5B/H3K4me3 through β-catenin/Myc, resulting in the promotion on cell proliferation, migration, and tumor growth of gastric cancer [41]. Another study indicated that the patients with high expression of NEK2 showed greater tumor depth, lymphatic invasion and peritoneal dissemination in colorectal cancer [42]. Moreover, NEK2 was overexpressed in metastatic cancers as well, including lymph node and liver metastases. Although NEK2 has been confirmed as an oncogene, which plays a critical role in a variety of cancers, the functional role of NEK2 in GBM has not been fully elucidated. In this study, we analyzed the transcriptome expression profiles of four published databases related to GBM and verified that the expression of NEK2 was significantly upregulated in GBM patients. Moreover, we identified that elevated NEK2 expression was closely related to poor prognosis in glioma patients. Next, molecular biological assays were performed to evaluate the potential role of NEK2 in GBM by lentiviral silencing and overexpression. The results showed that suppressing NEK2 attenuated the malignancy of GBM cells while NEK2 overexpression promoted the ability of migratory and invasive in GBM cells. Furthermore, the intracranial xenograft tumor model identified the promotion of NEK2 on the tumorigenicity of GBM cells in vivo. Additionally, high-throughput RNA sequencing, followed by KEGG analysis, GSEA analysis and GO analysis suggested that NEK2 was positively related to NF-κB signaling pathway. Subsequently, molecular biological assays proved that NEK2 was a critical regulator, which phosphorylated NIK and increased the activity and stability of NIK, thus inducing the activation of noncanonical NF-κB signaling pathway.

In resting cells, TRAF2 connects TRAF3 with the cellular inhibitor of apoptosis protein (cIAP)1/2 to form a TRAF3-TRAF2-cIAP1/2 multisubunit ubiquitin ligase complex [43]. TRAF3 does not have ubiquitinase activity. Instead, it functions as a bridge between cIAP1/2 and NIK, and recruits NIK to the complex [43]. Next, K48-linked ubiquitin chain mediated by cIAP1/2 leads to the proteasome-dependent degradation of NIK, which maintains NIK at a low level [44]. Upon receptor stimulation, TRAF3-TRAF2-cIAP1/2 complex raised to active receptors and the target of cIAP1/2-mediated K48 ubiquitination and proteosome-dependent protein degradation changes from NIK to TRAF3, resulting in the degradation of TRAF3 and the accumulation NIK [44]. After that, the downstream kinase IKKα is phosphorylated and activated by NIK, which then phosphorylates several residues on the C-teminus of p100. Phosphorylated p100 is recognized by the E3 ubiquitin ligase βTRCP and triggers the proteolytic processing of p100 into p52 [45]. Subsequently, the transcriptionally active RelB/p52 heterodimers translocate into the nucleus and regulate the expression of related genes. Although in previous studies, NF-κB signaling pathway has been identified to be involved in the progression of GBM, the correlation of NF-κB and NEK2 has not been assessed. Our data indicated that NEK2 knockdown reduced the protein levels of p52, while noncanonical NF-κB signaling pathway is mediated by RelB-p52 heterodimers whose activation are dependent on NF-κB-inducing kinase (NIK). Therefore, we hypothesize that NEK2 activates noncanonical NF-κB signaling pathway by phosphorylating NIK. To this end, GST pull down and CO-IP assays were performed. As expected, western-blot assays exhibited the presence of NEK2 and NIK in the affinity-purified protein complex, indicating that there was an interaction between NEK2 and NIK. Moreover, we observed that the phosphorylation of NIK was significantly decreased in NEK2 knockdown GBM cells. Consistently, the protein levels and the protein stability of NIK were reduced in NEK2 knockdown GBM cells when compared with control GBM cells. Taken together, our data so far above demonstrate that NEK2 induces the activation of noncanonical NF-κB signaling pathway by phosphorylating NIK and increasing the activity and stability of NIK. These findings for the first time reveal a new mechanism through which NEK2 leads to the malignant transformation of GBM, thereby helping to promote the design of more effective NEK2-targeted therapies for GBM.

Although the potential role of NEK2 in GBM and its related pathways have been well discussed in this study, further research on the molecular mechanism are still required for evaluating the clinical significance of NEK2 in GBM. Chen YW et al. reported that microRNA-128-3p overexpression inhibits breast cancer stem cell characteristics by downregulating NEK2 [46]. Another study identified miR-486-5p as an upstream regulator of NEK2 expression in hepatocellular carcinoma (HCC), thereby potentially opening a future therapeutic avenue to alleviate HCC progression [47]. However, the upstream regulators of NEK2 in GBM remain largely unknown. Although the direct interaction between NEK2 and NIK has been verified, whether NEK2 acts on other NIK regulatory proteins, especially TRAF3 is yet to identify. Also, further molecular experiments are needed to discover the phosphorylation sites through which NEK2 phosphorylates NIK. In addition, due to the heterogeneity of GBM, inhibition of a single candidate biomarker might bring unpredictable effects. Therefore, it is essential to evaluate GBM patients through a more comprehensive strategy before clinical management.

Overall, we found that NEK2 was significantly overexpressed in GBM and the patients with higher expression of NEK2 exhibited a poorer prognosis. Moreover, suppressing NEK2 attenuated the malignancy of GBM, while NEK2 overexpression promoted the ability of migratory and invasive in GBM. In addition, both bioinformatic and functional analysis confirmed that NEK2 was positively related to NF-κB signaling pathway. We further identified that NEK2 phosphorylated NIK, which increased the activity and stability of NIK, thereby inducing the activation of the noncanonical NF-κB signaling pathway. These results indicated that NEK2- NF-κB axis could be a potential drug target for GBM.

## Supplementary information

checklist

## Data Availability

The authors declare that all data in the article is available.
